# Effect of *Andrographis paniculata* and *Melia azadirachta* against the *Dengue virus*

**DOI:** 10.6026/973206300210730

**Published:** 2025-04-30

**Authors:** Kamalraj Mohan, K.P Gautam Srinivas, Jeevan Malaiyan, Suresh Arumugam

**Affiliations:** 1 Department of Microbiology, Meenakshi Medical College Hospital & Research Institute, Meenakshi Academy of Higher Education and Research (Deemed to be University), Kanchipuram-631552, Tamilnadu, India; 2 Department of General medicine, Meenakshi Medical College Hospital and Research Institute, Meenakshi Academy of Higher Education and Research, Tamilnadu, India; 3 Department of Microbiology, Annaii Medical College Hospital and Research Institute, Pennalur, Sriperumbudur, Chennai-602117, Tamilnadu, India; 4 Meenakshi Medical College Hospital & Research Institute, Meenakshi Academy of Higher Education and Research (Deemed to be University), Kanchipuram-631552, Tamilnadu, India

**Keywords:** Dengue, anti-viral activity, cytopathic effects (CPE)

## Abstract

Dengue infection is emerging as a global threat due to increased mortality and morbidity, especially in tropical and sub-tropical regions. Therefore, it is of interest to evaluate the antiviral activity of *Andrographis paniculata* and *Melia azadirachta* against *Dengue virus*-1 in a vero cell line using a cytopathic effects (CPE) reduction assay and cytotoxicity assessed by MTT assay. GC-MS analysis was conducted to identify phytochemicals in the plant extracts. Both extracts showed potent antiviral activity at 150 µg/ml and were non-toxic up to 1.5 mg/ml, with 100% cell viability. These findings suggest that *A. paniculata* and *M. azadirachta* are promising candidates against *Dengue virus*-1, warranting further investigation into their active compounds.

## Background:

Dengue is regarded as one of the viral illnesses that humans contract from arthropods. The most frequent vector that spreads the *Dengue virus* is Aedes aegypti. Classical dengue fever (DF), dengue hemorrhagic fever (DHF), and dengue shock syndrome (DSS) are the three categories into which the WHO has classified the *Dengue virus* [1]. Classical dengue fever typically produces a first infection and manifests as a mild febrile illness. However, in a small number of patients, the original infection progresses to DHF and DSS, which can be fatal [2]. Due mostly to dengue hemorrhagic fever, the occurrence of dengue infection raises the death and morbidity rate, particularly in Asian nations. Four serotypes of *Dengue virus*es, including DENV-1, DENV-2, DENV-3, and DENV-4, are members of the flaviviridae family. Over the past 20 years, DENV 1 and DENV 3 had more serious problems than DENV 2 and DENV 4 [3]. *Dengue virus*es comprise three structural proteins (capsid, pre-membrane, and envelope) and seven non-structural proteins (NS1, NS2A, NS2B, NS3, NS4A, NS4B, and NS5). The World Health Organization (WHO) describes dengue as a feverish disease primarily affecting adolescents, young children, and newborns. The traditional symptoms may show up three to fourteen days following an infectious mosquito bite. With symptoms ranging from mild to high-grade fever with severe headache, retro-orbital pain, rashes, and muscle and joint pain, it is not directly transmissible from person to person1. Since there is currently no effective vaccination to prevent infection, early detection and diagnosis are vital for affected patients [4]. There are fewer ways to detect dengue viral infection, including the Rapid Card Test, the MAC-ELISA method for Anti-IgM and IgG, viral isolation techniques, and molecular methods [5]. Patients with symptoms were treated with supportive care and hydration replenishment because there was no licensed vaccination [6]. Many investigations on medicinal plants are currently being conducted to generate the more recent anti-dengue medication. Extracts from medicinal plants and the bioactive substances they contain have strong antiviral properties against the *Dengue virus* [71]. Therefore, it is of interest to screen the anti-dengue properties of Indian medicinal herbs such as *Andrographis paniculata*, *Melia azadirachta*, *Carica papaya*, and *Aadadhoda vasica*.

## Materials and Methods:

The WHO guidelines for the selection of dengue cases were used. Each patient was given a preliminary form with the following details and their informed consent. Samples were collected at Sri Muthukumaran Medical College Hospital and Research Institute in Chennai, India, with institutional ethics approval (Human Ethics No: 46/IEC/23.12.2016). After onset, samples were taken on days 1 through 7 (mean = 3 days) and kept at -86°C.

## Extraction and amplification:

140 µl of RNA was extracted from the serum sample using the QIAamp viral RNA micro kit (Qiagen, Catalogue No. 52904) in accordance with the manufacturer's instructions. RNA is quantified using Nanodrop 2000 (Thermo Scientific Inc.), and viral RNA is converted into cDNA in accordance with the manufacturer's procedure for the Omni script RT kit (Qiagen, Catalogue No: 205111). Certain primers will be used to amplify a 511 bp area of the C-prM gene, in accordance with Fatima Z *et al.* (2011). A reaction mixer containing 2 µl 10 x PCR Buffer, 1 µl 500 µM dNTPs, 2.4 µl MgCl2, 1 µl 20 pM forward & reverse primer each, 5.6 µl d.H2O, and two units of Taq-DNA polymerase enzyme (Invitrogen Biotechnologies USA) was used to amplify the cDNA. Lastly, 5 µl of cDNA product was used. Denaturation at 94°C for 2 minutes is the first step in the PCR process for converting cDNA. This is followed by 35 cycles of denaturation at 94°C for 45 seconds, annealing at 52°C for 45 seconds, and extension at 72°C for 2 minutes. Ten minutes of a final extension will be done at 72°C. Likewise, cDNA conversion occurs after the second PCR round, with the exception that the annealing temperature is changed to 54°C for 45 seconds. Ultimately, 1.2% agarose gel will be used to present the amplified products, followed by ethidium bromide staining and visualization using a trans-illumination [8].

## Vero cells maintenance and collection:

Vero cells were acquired from Pune, India's National Center for Cell Science (NCCS). The minimum essentials medium (Sigma-Aldrich, Catalogue No. M4655) must be used to cultivate Vero cells. It must contain 10% fetal bovine serum (FBS), 2 mM L-glutamine, and antibiotics such as streptomycin (100µg/ml) and penicillin (100 IU/ml). Finally, it must be incubated at 37°C in a humidified environment with 5% CO2. Vero cells were treated with trypsin EDTA for five minutes at 37°C after being rinsed with PBS to ensure confluent growth [9].

## Virus isolation:

After sterilizing the serum sample using a 0.2µm syringe filter, the *Dengue virus* was isolated and used to infect Vero cells that were kept in Minimum Essentials Medium (Sigma-Aldrich, Catalogue No. M4655) that contained 2% FBS, 2 mM L-glutamine, 100 IU/ml penicillin, and 100µg/ml streptomycin (Hi-media, Catalogue No. A001A). When the cytopathic effects (CPE) became noticeable, the cells were extracted after being cultivated for 7-10 days at 37°C with 5% CO2. After being aliquoted, the culture harvests were kept at -86°C until they were further processed. Every tissue culture that tested negative for CPE was exposed to two blank cultures.

## Quantification of virus:

Tissue culture infectious dose (TCID50) assay by endpoint dilution assay was used to quantify the *Dengue virus*. In summary, 0.2 ml of the vortexes virus isolate was moved to the first tube to provide a 1/10 dilution, and 1.8 ml of MEM was added to tubes 1 through 7. After thoroughly mixing the tube, the serial dilution was carried out until the dilution reached 10-7. The 96-well ELISA plate was filled with 100 µl of each of the dilutions ranging from 10 -1 to 10-7. 100 µl was applied to each well using a dropper after cell suspensions containing roughly 1.5 x 105 cells/ml were produced. After adding 100 µl of cells and 100 µl of 2% MEM to cell control wells, the cells were incubated in a CO2 incubator at 37°C. Day 5 and Day 7 saw the examination of the plates, and CPE was noted. According to Karber's formula, TCID50 per 100 µl of viral solution was determined using L-d (S-0.5) [10, 11].

## Preparation of leaf extract:

Medicinal plants such as *Andrographis paniculata* (*A. paniculata*), *Melia azadirachta* (*M. azadirachta*), *Carica papaya* (*C. papaya*) and *Aadadhoda vasica* (*A. vasica*) were collected. from Periyapalayam Village, Tamilnadu, India (Figure 1 - see PDF). Taxonomic identification & authentication were obtained from the Department of Pharmacognosy, Siddha Central Research Institute, Anna Government Hospital, Arumbakkam, Chennai. Mature greenish leaves were washed in sterile water, shade-dried, powdered & kept at room temperature for further use. Extract preparation was performed according to the standard method by Shameel *et al.* [12].

## Anti-viral activity by CPE reduction assay:

Vero cells were cultured on 24-well plates at 37°C with a density of 2x106 cells per well, 5% CO2, and 10% Minimum Essentials Medium in a humidified environment. Following a 24-hour period, an 80% confluent monolayer was infected with 50µl of *Dengue virus* (5000 TCID50) and incubated for 90 minutes at 37°C with 5% CO2. The filtered extracts were applied to infected wells at varying concentrations (25µg/ml to 200µg/ml) and cultured for 10 days at 37°C in an atmosphere with 5% CO2. Every day, plates were inspected under a microscope to check for a decrease in the cytopathic effect (CPE). Both viral and cell control were preserved [13].

## MTT assays to measure the toxicity of extract:

The toxicity of extracts was screened by the MTT [3-(4, 5-dimethylthiazol-2-yl)-2, 5-diphenyltetrazolium bromide] assay under standard protocol [14]. Vero cells were cultured at 37° C on 12-well plates with a density of 2x106 cells per well, 5% CO2, and 10% Minimum Essentials Medium in a humidified environment. Extracts were applied to monolayers with 70% to 80% confluency after 24 hours, resulting in final concentrations ranging from 25 µg to 10 mg/ml. The plates underwent three more days of incubation in the previously described conditions. Each well was filled with 200 µl of MTT (Sigma-Aldrich, Catalogue No.M2003) solutions (5 mg/ml in phosphate buffer), which were then incubated for four hours at 37°C. After decanting the MTT solution, 250 µl of DMSO was added to each well, and formazan was removed from the cells. A 12-well ELISA plate reader was used to quantify color at 550 nm. A 1% Triton X-100 toxicity control was employed (Qualigens, cat. No. 10655). Every assay was carried out three times.

## Photochemical analysis by GC-MS:

*A. paniculata*, *C. papaya*, *A. vasica*, and M. azedica extracts were subjected to phytochemical analysis at Hubert Enviro Care Systems Pvt Ltd in Chennai, Tamilnadu, India. To identify the main components, gas chromatography-mass spectroscopy (GC-MS) was used [15]. One microliter of the methanolic extract of each of the four plants was injected into a GC-MS (JEOL GC mate) device to identify the chemicals. The main compounds were then assessed 40 minutes later.

## Results:

Patients with acute dengue infections exhibiting high-grade fever symptoms were found to have the *Dengue virus*. An IgM antibody specific to dengue was detected. Several continuous cell lines were employed to detect DENV proliferation and isolation. Vero cells were determined to be the best appropriate cell line for DENV isolation out of all those that were used. Vero cells were exposed to serum samples, incubated for 7-10 days at 37°C with 5% CO2, and their morphological alterations were monitored. CPE was shown to be typical of DENV.

Methanolic extracts of *A. paniculata*, *M. azadirachta*, *C. papaya* and *A. vasica* ranging from 25 µg/ml to 200 µg/ml were used to treat Vero cells infected with a *Dengue virus* with a 5000 TCID50. The viral replication was suppressed by *A. paniculata* and *M. azadirachta* at a dose of 150 µg/ml (Figure 2 - see PDF). CPE was prevented from developing after seven days of continuous therapy. In order to prevent the development of viral CPE in Vero cells, it seems that the extracts must be continuously present in the cell culture media. The lack of CPE and the blockage of virus multiplication by negative RT-PCR served as the foundation for this observation. When CPE was treated with different extracts, no inhibition was seen. The cytotoxic capability of the extract was assessed treatment-induced vero cell viability. Vero cells underwent a 72-hour incubation period after being exposed to different quantities of *A. paniculata* and *M. azadirachta*. The MTT assay was used to measure the cells' percentage vitality every 24 hours. Upon 72 hours, doses ranging from 25 µg/ml to 1.5 mg/ml demonstrated 100% vitality, as illustrated in Figure 3 (see PDF). From 2 mg/ml to 10 mg/ml, there was a slight cytotoxic effect. However, none of these concentrations showed 100 % toxicity.

Initial screening revealed the presence of alkaloids, flavonoids, saponins, sugars, phenols, steroids, tannins, diterpenes, and glycosides. The extracts of *A. paniculata* and *M. azadirachta* were subjected to GC-MS analysis, which revealed several chemicals, with a select handful being shown to be predominant. One of the substances, lupeol (chemical formula C30H50O; molecular weight 426.4 g/mol), displayed a peak area with RT of 30.18 in *A. paniculata* (Figure 4 (see PDF) and Figure 5 (see PDF)). In *M. azadirachta*, 17-Chloro-7-heptadecyne (chemical formula C17H31Cl; molecular weight 270 g/mol) displayed a peak region with RT of 28.3. This substance may be the cause of the observed antiviral action and was most prevalent in the extracts of *A. paniculata* and *M. azadirachta*.

## Discussion:

Dengue was regarded as a serious health issue since the vaccine failed to provide prophylactic. There is currently no data on medications that combat the *Dengue virus* [16]. Globally, however, there have been documented cases of dengue recurrence and its complications [17]. The present circumstances have justified the need for efficient medications to prevent dengue illness. With this idea in mind, we investigated the anti-*Dengue virus* properties of two Indian medicinal plants, *A. paniculata* and *M. azadirachta*, which are known to have a variety of different medical uses. *A. paniculata* methanolic extract was used in the experiments to demonstrate the anti-dengue action using in silico analysis and the CPE reduction assay [18-19]. According to one study, an aqueous extract from fresh green leaves of *M. azedarach* exhibits exceptional antiviral activity, preventing the in vitro replication of many DNA and RNA viruses [20]. We employed strains of the *Dengue virus* isolated from individuals with acute dengue-1 infection who also tested positive for anti-dengue IgM antibody because standard strains were not available for testing. 150 µg/ml of *A. paniculata* and *M. azadirachta* methanolic extracts inhibited dengue-1 viral replication and prevented approximately 95% of CPE development when exposed to extract for 7 days, according to antiviral assays conducted with different concentrations of these extracts. This was confirmed by negative RT-PCR results. The toxicity of the extract was examined using the MTT test. Cell viability was 100% and the extracts were confirmed to be non-toxic up to a concentration of 1.5 mg/ml. Furthermore, it is commonly known and accepted that the bioactive Phytocomponents present in plants are what give them their medicinal potential. The andrographolide from *A. paniculata* was tested against DENV serotype 2 in two cell lines (HepG2 and HeLa), whereas the activity against DENV 4 was tested in one cell line (HepG2), according to Panraksa *et al.* (2017) [21]. The findings demonstrated that andrographolide significantly inhibited DENV in both cell lines, lowering the incidence of viral replication and cell infections. DENV 2's effective concentrations (EC50) for HepG2 and HeLa were 50% [22]. This study used the Vero cell line to assess anti-dengue activity. Through the prevention of CPE production rather than viral multiplication, the methanolic extract of *A. paniculata* at its MNTD exhibits anti-dengue 1 inhibitory activity [23]. This study showed that *A. paniculata* and *M. azadirachta* inhibited DENV-1 replication at a dose of 150 µg/ml, with negative RT-PCR results. *A. paniculata* and *M. azadirachta* Phytocomponents active principles were examined in this work and the plant extract's antiviral activity against *Dengue virus*-1 was evaluated at varying extract concentrations to determine the most effective ones.

## Conclusion:

Natural compounds are safe and non-toxic compared with synthetic agents. *A. paniculata* and *M. azadirachta* showed antiviral properties against *Dengue virus* -1 with less cytoxicity. Further, investigation is essential to isolate and purify the active compounds in order to develop the potential anti-dengue drug for therapy.
